# Diatom Analysis in Drowning: A Critical Review of Reliability, Contamination, and Medico-Legal Interpretation

**DOI:** 10.7759/cureus.100870

**Published:** 2026-01-05

**Authors:** Abdullah S Al-Najjar, Faisal N Shata, Omar Ba Mhel, Yamen Gutob, Mohammed Alhazmi, Zayed Alharbi, Rami A Choghari, Aseel A Qashar, Basem Basudan, Naif Aljohani, Abdulkreem Al-Juhani

**Affiliations:** 1 Medicine and Surgery, Umm Al-Qura University, Makkah, SAU; 2 College of Medicine, Umm Al-Qura University, Makkah, SAU; 3 Urology, Umm Al-Qura University, Makkah, SAU; 4 Forensic Medicine, Riyadh Forensic Medicine Center, Riyadh, SAU; 5 Forensic Medicine, Forensic Medicine Center, Jeddah, SAU; 6 Surgery, King Abdulaziz University Faculty of Medicine, Jeddah, SAU

**Keywords:** contamination, diatom test, dna metabarcoding, drowning, evidence reliability, forensic pathology, medico-legal interpretation, microscopy

## Abstract

The diagnosis of fatal drowning remains one of the most challenging tasks in forensic pathology, as no single autopsy finding is pathognomonic, and interpretation relies on the integration of scene information, circumstances, and ancillary investigations. Among supportive tests, diatom analysis has been used for decades, yet its medico-legal value continues to be debated due to methodological heterogeneity, contamination risks, and inconsistent interpretive frameworks.

This review critically examines diatom evidence in drowning from a comparative and fit-for-purpose perspective, focusing on mechanistic plausibility, alternative non-drowning explanations, and methodological blind spots that undermine evidentiary reliability. Conventional microscopy-based diatom testing and emerging DNA-based and metagenomic approaches are compared with respect to what they detect, how contamination may arise, and how results are currently interpreted in forensic casework. Particular emphasis is placed on low-count diatom findings in closed organs, where recent evidence demonstrates substantial vulnerability to laboratory, consumable, and postmortem contamination.

Drawing on recent systematic syntheses, controlled postmortem studies, and newly identified contamination sources, this review argues that mechanistic plausibility does not equate to forensic reliability. Diatom findings are best interpreted as supportive evidence whose weight depends on explicit contamination control, transparent reporting, and alignment with a clearly defined medico-legal proposition. To address persistent comparability and interpretation gaps, a minimum reporting dataset, minimum contamination-control principles, and a decision-oriented interpretive framework are proposed.

In conclusion, diatom testing should neither be regarded as definitive proof nor dismissed outright. When applied selectively and interpreted within a contamination-aware, proposition-driven framework, diatom evidence may contribute meaningfully to drowning diagnosis and drowning-site inference, while avoiding overstatement of its probative value.

## Introduction and background

Medico-legal "drowning" is not a single pathognomonic autopsy finding but a conclusion reached by integrating scene reconstruction, circumstances, and autopsy/laboratory findings, particularly because many classical findings are nonspecific, can be absent, or can be confounded by immersion artifacts, resuscitation, and decomposition [1,2]. Autopsy practice guidelines for bodies recovered from water explicitly emphasize structured sampling, documentation, and multi-source interpretation precisely because submersion may be incidental to death, postmortem, or contributory but not primary [2]. Contemporary reviews continue to describe drowning diagnosis as one of the most difficult tasks in forensic pathology because the strongest indicators (e.g., froth/airway findings, emphysema aquosum, watery fluid in sinuses, and other ancillary markers) are variably present and variably specific across contexts (freshwater vs seawater, time in water, decomposition stage, and medical interventions) [1,2].

The diatom test, classically the detection and sometimes taxonomic comparison of diatoms in tissues and or fluids, has historically been used as supportive evidence rather than a standalone determinant of drowning, and its medico-legal value remains debated [3-5]. Recent forensic literature frames diatom findings as an adjunct that may contribute to drowning diagnosis and or drowning site inference when interpreted alongside conventional autopsy findings and scene information, while simultaneously warning that the evidentiary weight is undermined by nonstandardized protocols and contamination/false-positive pathways [3-5]. A systematic review focused on medico-legal interpretation concludes that, despite longstanding use, the evidentiary value of diatom testing for diagnosing drowning continues to be "elusive and contentious" because interpretation is inseparable from methodological heterogeneity and contamination risk [3].

The core controversy is not merely whether diatoms can be found in drowned bodies, but whether a given diatom finding (organ type, quantity, species pattern, and match to reference water) is sufficiently reliable to shift the probability of drowning versus postmortem immersion or alternative causes of death [3,4]. The stakes are practical and legal: misclassification of drowning can misdirect investigations, distort cause-of-death certification, and affect criminal/civil outcomes (e.g., homicidal staging versus accident, insurance, and liability contexts) [1-3]. Because autopsy guidelines emphasize a disciplined, evidence-graded approach for bodies recovered from water, any ancillary test that is vulnerable to false positives or laboratory artifacts can disproportionately influence medico-legal conclusions if its limitations are not transparently controlled and reported [2,3].

## Review

Methodological approach and scope of the review

This work was designed as a narrative, critical, and comparative review addressing the medico-legal value of diatom evidence in the diagnosis of fatal drowning and drowning-site inference. Rather than conducting a formal systematic review with meta-analysis, the objective was to critically evaluate mechanistic plausibility, methodological variability, contamination vulnerabilities, and interpretive frameworks that shape the evidentiary weight of diatom findings in forensic practice.

The literature was identified through targeted searches of major biomedical and forensic databases, including PubMed/MEDLINE, Scopus, and Web of Science, supplemented by manual screening of reference lists from key review articles and guideline documents. Searches focused on combinations of keywords related to drowning, diatom testing, forensic pathology, microscopy-based diatom analysis, DNA/metabarcoding approaches, contamination, and drowning-site inference. Particular emphasis was placed on recent literature (approximately 2022-2025) to capture methodological developments, newly identified contamination sources, and contemporary medico-legal perspectives.

The included sources comprised systematic reviews, narrative reviews, controlled post-mortem studies, experimental investigations, methodological papers, and authoritative professional guidelines relevant to forensic drowning diagnosis. Case reports and single-case descriptions were considered only when they illustrated specific methodological or interpretive issues. No formal quantitative pooling or statistical synthesis was attempted, given the substantial heterogeneity in study design, analytical protocols, outcome measures, and reporting standards across the literature.

The retrieved evidence was analyzed using a comparative framework, contrasting conventional microscopy-based diatom testing with DNA-based and metagenomic approaches. Particular attention was paid to points in the analytical workflow where false-positive findings may arise, the interaction between postmortem change and diatom detection, and the implications of nonstandardized methods for medico-legal interpretation. On this basis, the review proposes a fit-for-purpose interpretive model, including minimum reporting elements and contamination-control considerations, intended to support transparent and defensible forensic conclusions.

Mechanistic basis and competing explanations

How Diatoms Are Hypothesized to Reach “Closed Organs”

The classical mechanistic narrative underpinning forensic diatomology is that aspiration of diatom-containing water during drowning introduces diatoms into the lungs, followed (in some accounts) by translocation across the alveolar-capillary interface into the circulation and subsequent distribution to “closed” organs such as liver, kidney, brain, and bone marrow. [3,4,6,7] This mechanism is frequently invoked to justify why detection of diatoms in closed organs has been interpreted as more supportive of antemortem aspiration than diatoms confined to airway/lung compartments alone. [3,4,7] However, recent reviews underscore that mechanistic plausibility does not automatically translate into forensic reliability, because the diagnostic problem is inherently comparative: how often similar findings occur via non-drowning routes or contamination, and under what procedural conditions. [3,4]

Alternate Routes and the Problem of “Background” Diatoms

Ante-mortem inhalation/ingestion and background presence. Diatom detection is complicated by the ubiquity of diatoms in aquatic environments and by the realistic possibility that small numbers may be encountered in non-drowning contexts, including background exposure or pre-existing presence in airway/gastrointestinal compartments. [3,6,7] Controlled postmortem evaluations and the broader forensic literature acknowledge that “false-positive” diatom findings, defined as diatom detection in individuals not dying from drowning, are a central interpretive vulnerability, and they may involve both ante-mortem and postmortem pathways. [3,6,7] Postmortem passive entry during immersion and handling; Bodies recovered from water may have experienced variable postmortem immersion intervals, handling, and environmental movement, all of which can introduce or redistribute water and particulate material in or on the body. [1,2] Because drowning diagnosis already requires careful separation of true drowning physiology from immersion artefacts, any particulate-based marker (including diatoms) must be assessed against the baseline possibility of passive entry or redistribution after death. [1-3] Laboratory contamination and procedural artefacts; A critical competing explanation-highlighted across recent systematic and narrative reviews-is that diatom “positivity,” particularly at very low counts, can be generated during autopsy sampling, tissue processing, or slide preparation rather than reflecting a biological translocation pathway. [3-5,7] Empirical studies demonstrate that contamination can originate from underestimated sources such as reused digestion labware (e.g., Kjeldahl flasks used in strong-acid protocols), where adherent frustules persist and later release into subsequent samples. [7] A 2025 report further identifies disposable microscope slides as an unreported contamination source, warning that even ostensibly sterile single-use consumables can introduce diatoms into preparations. [5]

Setup for the Review’s Comparative Argument

Across these competing explanations, an important forensic principle emerges: mechanistic plausibility ≠ evidentiary reliability [3,4]. Even if translocation to closed organs occurs in true drowning, the medico-legal question is whether the observed diatom signal (organ distribution + quantity + species pattern + match to reference water) is sufficiently robust against realistic contamination and non-drowning pathways to warrant diagnostic weight [3,4,7]. This review therefore treats diatom evidence as a comparative problem in which the “signal” must be evaluated relative to protocol-dependent noise, environment-dependent baseline exposures, and reporting practices that determine reproducibility and courtroom defensibility [3-5,7].

Comparative methods

Why a Method-Comparison Frame Is Essential

The diatom debate persists largely because different laboratories (and different publications) are often not talking about the same analytic object. Microscopy typically documents siliceous valves/frustules as physical trace evidence after digestion/filtration, whereas molecular approaches detect diatom DNA (amplicons or metagenomic reads) whose persistence, transport, and contamination dynamics differ from intact frustules [3,4,8-11]. Because the evidentiary meaning depends on what is being detected, where it is detected, and how contamination control is performed, method comparison is not an academic exercise; it is the prerequisite to interpreting discordant “positivity” rates across studies and laboratories [3,4,7,11].

Conventional Microscopy-Based Diatom Testing: Sample Choice, Digestion, and Quantification Approaches

Methodological considerations begin with sample choice. Conventional protocols typically prioritize lung tissue and may also include closed organs such as liver, kidney, bone marrow, and blood, with the closed-organ strategy intended to increase specificity beyond airway-only detection [3,4,7]. However, interpretation of closed-organ positivity is especially sensitive to contamination and protocol variability because the expected diatom burden is often far lower than in lungs or site water, thereby magnifying the impact of low-level artifacts [3,7]. Digestion and isolation methods classically involve dissolving organic tissue using strong acids or proteolytic enzymes, followed by concentration and visualization of diatom frustules with light microscopy and/or SEM [4,7,8]. Strong-acid digestion is widely characterized as hazardous and technically demanding due to violent reactions, spatter risk, and strict glassware requirements, whereas enzymatic approaches are framed as potentially safer and, depending on implementation, gentler on frustules [4,8]. Quantification remains a major source of interstudy disagreement, particularly regarding whether results are treated as binary or quantitative, and if quantitative, how thresholds are defined and whether they are organ-specific [3,4,7]. A systematic review of medico-legal interpretation stresses that the absence of standardized concentration reporting and validated thresholds undermines cross-study comparability and promotes circular reasoning when diatom findings are used both to define drowning cases and to “validate” the test itself [3].

Key Methodological Forks That Drive False Positives

The microscopy-based workflow contains multiple “forks” that can generate false positives or inflate evidentiary confidence if not explicitly controlled and reported [3-5,7]. The most practically demonstrated forks include, first, the history and reuse of labware (such as strong-acid digestion). Reuse of Kjeldahl flasks can seed subsequent samples with adherent diatoms, producing false-positive counts that persist despite cleaning, likely because frustules (silica-based) can adhere or partially fuse to glass surfaces and later release [7]. Second, slide/consumable contamination. Even disposable microscope slides may contain diatoms, introducing contamination at the final analytic stage and creating a misleading impression of tissue-derived diatoms [5]. Third, autopsy sampling cross-contamination. Because bodies recovered from water may have diatoms on the skin, clothing, airway surfaces, or within luminal compartments, organ sampling and instrument handling can introduce diatoms into closed-organ samples unless strict barriers and a sequential sampling approach are used [2,3,7]. Fourth, low-count interpretive vulnerability. When expected true-positive burdens are low, particularly for closed organs, even a few contaminant diatoms can drive a “positive” classification, making chain-of-custody, blanks, and negative controls central to interpretation rather than optional laboratory good practice [3,5,7].

Why Standardization Is Still Not Solved

Despite decades of use, standardization remains unresolved because published and operational protocols differ in organ selection, digestion chemistry/enzymes, filtration and concentration steps, microscopy platform and taxonomic rigor, quantitative metrics and thresholds, and quality-control and contamination reporting [3,4,7]. Both the systematic review and the recent narrative review explicitly argue that these variations prevent stable estimates of sensitivity/specificity and make it difficult to assign consistent medico-legal weight to diatom findings across jurisdictions [3,4]. The discovery of previously underappreciated contamination sources (reused digestion glassware and disposable slides) further supports the argument that standardization must extend beyond “which organ and which acid” to include verified clean consumables, negative controls, and contamination audits at every stage [5,7].

DNA/metabarcoding/metagenomic diatom approaches

*What Molecular Diatom Approaches* *Detect (DNA) vs What Microscopy Detects (Valves/Frustules)*

Molecular diatom approaches target nucleic acids, either via targeted amplification (DNA barcoding/metabarcoding) or broader metagenomic strategies, whereas microscopy-based testing documents siliceous frustules/valves as intact physical structures following tissue digestion [8,10,11]. This distinction matters for forensic inference: DNA-based detection may succeed when frustules are few, fragmented, or difficult to identify morphologically, but it also introduces new vulnerability to molecular contamination, amplification bias, and misassignment from reference libraries [8,11]. Conversely, frustule visualization provides a tangible morphological object but is constrained by taxonomic expertise demands and by the same contamination risks that can introduce frustules from laboratory materials [4,5,7].

Strengths and Weaknesses in a Forensic Context

Strengths: DNA-based workflows can support high-throughput profiling, can operate with small sample volumes, and may facilitate drowning-site inference by comparing assemblage signatures across locations or water types [8,9,11]. For example, metagenomic diatom analysis has been evaluated for geographic/watershed inference across numerous water samples and showed that some distinctions, such as freshwater versus seawater patterns, may be feasible even when precise geographic assignment remains difficult [8]. Targeted DNA barcode work in a forensic framing highlights metabarcoding as a route to “forensic discrimination” while also emphasizing that careful marker/primer selection is essential [11].

Weaknesses: Molecular diatom evidence is constrained by incomplete or unreliable reference libraries, mislabeled sequences in public repositories, amplification and primer bias, and the need for transparent bioinformatic thresholds and reporting standards if results are to be reproducible across laboratories [11]. In a forensic setting, these weaknesses directly affect admissibility and interpretive confidence because the chain from read assignment to taxon to environmental inference must be auditable [8,11]. In addition, metagenomic applications show that large-scale sampling can still fail to produce stable clustering by watersheds or rivers, underscoring that ecological similarity and temporal variation may blur geographic inference even when sequencing works technically [8].

Where They Cannot Replace Microscopy Yet

Current literature positions molecular methods as complementary rather than fully substitutive for microscopy in medico-legal drowning diagnosis for several reasons. First, reference libraries and taxonomic ground truth are limited. DNA barcoding work explicitly identifies the absence of consensus on diatom barcodes and the urgent need for correctly identified reference sequences as barriers to routine forensic deployment [11]. Second, thresholds and courtroom-ready reporting remain underdefined. The medico-legal systematic review stresses that evidentiary value depends on standardization and interpretation frameworks; molecular data amplify this need because bioinformatic thresholds can shift conclusions without visible morphological anchors [3,11]. Third, cross-laboratory reproducibility is not yet demonstrated at the level required for routine forensic diagnostics. Both the narrative and systematic reviews emphasize heterogeneity as a central problem, and molecular methods add additional steps, including extraction, amplification, sequencing platform, and pipeline choices, that can vary across laboratories [3,4,11]. Fourth, contamination risks persist but change form. Whereas microscopy is vulnerable to frustule contamination from labware and consumables, including new sources such as disposable slides, molecular workflows are vulnerable to DNA contamination and reference misassignment; both demand staged controls and transparent reporting [5,7,11]. Notably, novel microscopy-oriented innovations aim to reduce taxonomic burden and improve practicality, such as simplified optical classification frameworks and lossless extraction methods, suggesting that the replacement question may be less productive than defining evidence-grade roles for each modality within a harmonized, contamination-aware workflow (Table 1) [12-19].

**Table 1:** Side-by-side comparison of conventional microscopy vs DNA/metagenomic diatom methods

Domain	Conventional microscopy-based diatom testing	DNA/metabarcoding/metagenomic diatom approaches
What is detected	Physical frustules/valves (morphology-based evidence) [4,6]	Diatom DNA signal (sequence-based evidence) [10,11]
Typical samples	Lung, stomach contents, and—controversially—closed organs (e.g., liver/kidney/bone marrow) depending on proposition [3,4,14]	Similar tissues can be used, but interpretive link to “circulation of diatoms” is indirect because DNA ≠ frustules [10,11]
Core workflow	Tissue digestion → concentration/filtration → slide mounting → microscopic identification/counting [4,6,12]	DNA extraction → amplification (barcode) or shotgun sequencing → bioinformatics → taxonomic assignment [8,9,11]
Typical failure modes	Low-count findings dominated by contamination; identification variability; inconsistent quantification/thresholds across labs [3-5,7]	Database incompleteness/misannotation; variable pipelines; uncertain thresholds mapping reads to forensic propositions; contamination/cross-talk if workflow not segregated [8,11,20-22]
QC requirements (minimum)	Reagent blanks; equipment blanks; slide/consumable blanks where low-count claims matter; documentation of sample order and handling [3-5,7]	Negative controls across stages (reagent/extraction/PCR); segregation of incompatible activities; monitoring and documentation aligned with established contamination-control doctrine [21,22]
Interpretive pitfalls	“Rare diatoms in closed organs” may reflect contamination or post‑mortem effects; mechanism plausibility ≠ evidentiary reliability [3-5,14]	High sensitivity can detect background DNA; DNA presence does not automatically establish aspiration/circulation; cross-lab reproducibility is critical [8,10,11]
Where it is currently strongest	Supportive evidence (especially when combined with robust contamination controls and contextual findings) [3,4]	Research and adjunctive inference (e.g., community patterning for site questions) with ongoing validation needs [3,8,11]

The contamination problem

Contamination is the most destabilizing uncertainty in forensic diatom testing because it can generate the same low-level signal that is often interpreted as supportive of drowning: one or a few frustules/valves detected in deep tissues or “closed organs” [3,4,14]. This matters because the medico-legal debate is rarely about abundant diatoms in lungs; it is frequently about “rare diatoms” in tissues where they are presumed unlikely to appear without ante-mortem aspiration and circulation [3,4].

Autopsy Contamination vs Laboratory Contamination

A practical way to prevent interpretive drift is to treat diatom contamination as two linked, but distinct, risk domains: autopsy (pre-analytical) contamination and laboratory (analytical) contamination [3,4]. Autopsy contamination risks include cross-transfer from wet hair/skin/clothing, splash/aerosol transfer during evisceration, and instrument-to-organ carryover when sampling multiple organs without strict segregation [2-4]. On the other hand, laboratory contamination risks include vessel/equipment carryover, contaminated reagents or water used in digestion/rinsing, and transfer from laboratory consumables (including slides and coverslips) that contact the processed pellet or filtrate [3,4,6,7].

Slide/Reagent/Equipment Contamination (the “Silent” Chain)

Several lines of evidence indicate that very low diatom counts, the zone where case interpretation becomes contentious, are also the zone where contamination can dominate (Table 2) [3,7,14].

**Table 2:** False positive pathways and mitigation (with slide manufacturing contamination as a distinct row)

False‑positive pathway	Mechanism (how the signal is created)	Practical mitigation (minimum)	Key supporting sources
Postmortem immersion/passive entry	Waterborne material can enter open organs postmortem; severe postmortem change may increase detection in closed organs, complicating specificity [3,14]	Interpret open-organ findings cautiously; stratify by decomposition; prefer proposition-driven sampling and report limitations explicitly [3,4,14]	[3,4,14]
Autopsy sampling contamination	Cross-transfer between organs or from external surfaces during sampling and handling [2-4]	Dedicated instruments per organ or validated decontamination; strict sampling order; documentation of handling and barriers [2-4]	[2-4]
Lab contamination—equipment/vessels	Carryover from digestion vessels/processing equipment (e.g., re-used flasks) can introduce diatoms [7]	Single-use or dedicated vessels; batch blanks; documented cleaning validation [3,4,7]	[3,4,7]
Lab contamination—reagents/water	Contaminated reagents/rinse water can seed low-count diatoms [3,4,6]	Reagent blanks; validated water sources; aliquoting; batch tracking and lot recording [3,4,21,22]	[3,4,21,22]
Slide‑manufacturing contamination (2025)	Disposable slides themselves can carry diatoms, creating a false “organ” signal at the mounting stage [5]	Slide/consumable blanks; verified low-contamination consumables; documentation of slide lots; interpret low-count findings only with control data [3-5]	[3-5]

A classic example is the demonstration that re-used digestion vessels can serve as an underestimated “blind spot” source of diatoms during strong-acid workflows, producing notable false-positive patterns unless strict single-use/segregation is enforced [7]. Similarly, standardized postmortem studies have shown that false-positive diatom findings remain a real challenge even when protocols are controlled, highlighting that procedural discipline reduces but does not eliminate the contamination threat [6].

Decomposition and “Natural Load” as Contamination Amplifiers

Recent evidence indicates that severe postmortem change can be associated with higher diatom detection in closed organs, while resuscitation does not necessarily explain increased closed-organ diatom concentrations, supporting the possibility that postmortem factors and/or contamination contribute materially to the signal [14]. This creates a diagnostic trap: decomposition both increases interpretive uncertainty for drowning generally and increases vulnerability to low-level diatom findings that may be nonspecific or contamination-driven [1,2,14].

Key take-home is that contamination control is not “QC paperwork”; it is the core evidentiary safeguard.

Because systematic syntheses conclude that diatom evidence remains difficult to compare across studies and laboratories, contamination control is the single most immediately actionable lever to make diatom findings “fit for purpose” rather than rhetorically persuasive [3,4].

Drowning site inference: where the field is heading

Assemblage Matching (Microscopy-Forward Logic)

The classic site inference proposition is comparative; diatom assemblages recovered from tissues are compared with assemblages from suspected drowning media [4,18]. However, feasibility is constrained by spatiotemporal variability in diatom communities, overlap of taxa across habitats, and the need for robust sampling strategies and statistical comparators rather than anecdotal “species matches” [19,20].

Morphology With Routine Microscopy: Toward Feasible Workflows

A current direction is to use simplified morphology-based classification schemes under routine light microscopy as a pragmatic compromise between full taxonomic resolution and operational feasibility [13,20]. Pilot work suggests that simplified optical classification can support comparative inference under defined constraints, but it requires external validation, explicit error rates, and contamination-aware study designs before medico-legal deployment [3,4,13].

DNA/Metabarcoding/Metagenomic Inference-Promise With Forensic Friction

DNA-based approaches (metabarcoding/metagenomics) can, in principle, improve sensitivity and recover community patterns that are hard to resolve morphologically and have been explored explicitly for drowning-site inference [8-11]. Yet these approaches detect DNA, not necessarily intact frustules/valves, which complicates legal interpretation when the evidentiary narrative is framed around “physical diatoms transported by aspiration and circulation” [10,11]. Practical constraints include database completeness and curation, reproducibility across laboratories, and the need for agreed thresholds that map molecular reads to forensic propositions (e.g., drowning vs postmortem immersion; site A vs site B) [3,4,8,11].

Comparative evidence summary (narrative “results”)

Evidence Tiering Used for Adjudication

Because the literature is heterogeneous, a “strength-of-evidence” approach is more defensible than absolute statements (Table 3) [3,4].

**Table 3:** Comparative overview of diatom detection modalities and interpretive vulnerabilities (for drowning diagnosis and site inference)

Modality	Primary target (“what is detected”)	Typical output	Key strengths (for forensic use)	Dominant vulnerabilities/failure modes	Notes for medico-legal interpretation
Conventional microscopy-based diatom test	Intact frustules/valves (silica structures) recovered after digestion/filtration [4,7]	Presence/absence and/or counts; sometimes species comparison to site water [3,4]	Physical trace evidence; long forensic tradition; can support site inference when taxonomy and reference water sampling are strong [3,4]	Low-count false positives from labware/consumables; cross-contamination during autopsy; protocol heterogeneity; high expertise demand [3–5,7]	Treat as supportive; evidentiary weight is conditional on contamination controls and reporting of quantitative metrics and blanks [3,5,7]
Targeted DNA barcoding/metabarcoding	Amplified diatom DNA (marker-specific amplicons) [9,11]	Taxonomic list/relative abundance from sequencing reads [11]	High-throughput profiling; potential to compare assemblage signatures; can work when morphological ID is difficult [11]	Barcode/primer bias; incomplete/mislabeled reference sequences; contamination at the DNA level; threshold choices strongly affect outputs [11]	Requires auditable pipelines, curated references, and defined interpretive thresholds for forensic defensibility [11]
Metagenomic diatom analysis (e.g., 18S-based community profiling within a metagenomic framework)	Broader genetic signal from water/tissue extracts (eukaryotic 18S signal including diatoms) [8]	Community profiles + ordination/clustering for inference [8]	Can operate from small water volumes; evaluated for broad distinctions (e.g., freshwater vs seawater) [8]	Limited geographic resolution in large sampling; ecological similarity blurs clustering; still vulnerable to contamination and reference issues [8,11]	Promising for constrained questions (broad water-type discrimination) but uncertain for precise drowning-site assignment [8]

The evidence can be categorized into four tiers. Tier one (highest) includes systematic synthesis and multicase comparative human studies with controls and transparent methods. Tier two consists of controlled postmortem or experimental designs that isolate contamination and immersion effects. Tier three comprises single-center case series and methodological notes with limited controls or variable reporting. Tier four (lowest) is based on mechanistic plausibility arguments without evidentiary safeguards or validated thresholds [3,4,6,7,14,20].

“Pro vs Con” Statements and Adjudication

Pro statement A argues that diatoms in closed organs support antemortem drowning because circulation is required [4]; however, adjudication finds that although mechanistic plausibility exists, systematic evaluation shows conflicting data and significant contamination and comparability limitations that prevent closed-organ diatoms from serving as standalone proof [3,4]. Con statement A holds that low-count diatoms in closed organs are often nonspecific because contamination can mimic the signal [5,7], an assertion strongly supported by demonstrations of contamination from equipment and consumables and reinforced by evidence linking postmortem change to increased closed-organ detection consistent with postmortem effects and/or contamination [5,7,14]. Pro statement B suggests that microscopy-based counts can help separate drowning from postmortem immersion [4], but adjudication concludes that while potentially useful as supportive evidence, this approach requires standardized quantification, validated thresholds, and rigorous contamination controls, with systematic review indicating insufficient comparability for universal thresholds [3,4]. Pro statement C proposes that DNA-based diatom profiling improves sensitivity and may aid site inference [8,9], and adjudication supports this as a research direction but not a replacement for microscopy in medico-legal practice due to ongoing limitations in DNA signal interpretation, reference libraries, and cross-laboratory reproducibility [8,10,11]. The bottom line is that the debate is not whether diatoms are inherently good or bad, but which diatom results are fit for purpose under explicit contamination control and reporting standards (Table 4) [3,4,14].

**Table 4:** Evidence-threat matrix: competing explanations for diatom “positivity” and practical mitigation points

Competing explanation/contamination pathway	Where it arises in casework	Why it matters for interpretation	Representative evidence from recent literature	Mitigation/QA emphasis (comparative)
Background/ante-mortem presence (airway/GI exposure, low-level diatoms)	Prior to death; can persist into autopsy sampling [3,6,7]	Creates low-count positives in non-drowning contexts, especially if binary reporting is used [3,6,7]	False-positive concern is explicitly documented as a central reliability issue in postmortem studies and reviews [3,6,7]	Favor quantitative reporting; interpret low counts cautiously; integrate with full autopsy/scene context [3]
Postmortem passive entry/redistribution during immersion or recovery	After death, during immersion and body handling [1–3]	Can mimic drowning-related particulate presence and confound cause-of-death inference [1–3]	Reviews and guidelines emphasize immersion artefacts and the need for integrated diagnosis [1,2]	Structured scene + autopsy correlation; avoid over-weighting isolated diatom findings [1–3]
Autopsy sampling cross-contamination	During evisceration and organ sampling [2,3,7]	Can seed “closed-organ” samples with external/airway diatoms, inflating specificity claims [3,7]	Contamination during autopsy is recognized as a mechanism that limits reliability [3,7]	Sequenced sampling order, clean instruments, barrier techniques, documented controls [2,3]
Reused digestion labware (Kjeldahl flasks)	Strong-acid digestion workflow [7]	Produces reproducible false positives despite cleaning; disproportionately affects low-count closed organs [7]	Demonstrated false positives from reused flasks; authors recommend only new/unused flasks [7]	Single-use or verified-clean labware; staged negative controls; explicit labware history documentation [7]
Disposable microscope slides contamination	Slide preparation stage [5]	Introduces diatoms at the final step, creating persuasive but spurious “tissue” diatoms [5]	Newly reported contamination source in disposable slides [5]	Lot testing, slide blanks, procurement QA, document slide brand/batch [5]
Molecular contamination + reference misassignment	DNA extraction/sequencing + bioinformatics [8,11]	Can generate false taxon calls and misleading comparisons across sites/cases [11]	Mislabeling in public databases and lack of consensus barcodes highlighted as key limitations [11]	Curated reference libraries, negative controls, transparent pipelines, and thresholds [11]

Medico‑legal interpretation framework (unique contributions)

 A Minimum Reporting Dataset (MRD)

Given the documented comparability gaps and contamination vulnerability, a minimum reporting dataset is necessary for defensible interpretation and for future meta‑analysis [3,4]. The proposed MRD is provided as a journal‑ready checklist (Table 5) and is anchored in recurring deficiencies highlighted in systematic and critical reviews [3,4,20].

**Table 5:** Minimum reporting checklist

Item to report (minimum)	Why it must be reported	Source(s)
Case proposition (what question the test addresses)	Prevents “test-as-routine” misuse; aligns interpretation with a defined medico‑legal claim [3,4]	[3,4]
Organ(s) tested + rationale (open vs closed organs)	Closed-organ interpretation is controversial and more contamination‑sensitive, especially at low counts [3,4,14]	[3,4,14]
Tissue mass/volume analyzed per organ	Quantification is otherwise non-comparable across studies/labs [3,4]	[3,4]
Digestion method + parameters (reagent, time, temp)	Method choice affects recovery and damage; drives inter-lab variability [4,6,12]	[4,6,12]
Concentration/filtration steps + recovery documentation	Recovery losses can mimic “low diatom burden” and bias comparisons [4,12]	[4,12]
Microscopy identification level (genus/species) + criteria	Taxonomic variability is a known source of inconsistency; criteria must be explicit [3,4,11]	[3,4,11]
Quantification metric (e.g., diatoms per gram) + counting rules	Without defined rules, thresholds and comparisons are invalid [3,4,14]	[3,4,14]
Water sampling strategy (site, timing, replicates)	Site inference requires comparable environmental references; diatom communities vary [4,19,20]	[4,19,20]
Contamination controls: reagent blanks + equipment/vessel blanks	Equipment/reagents are documented contamination sources; blanks are essential [3,4,6,7]	[3,4,6,7]
Contamination controls: slide/consumable blanks when low-count claims matter	Disposable slides can be contaminated; control data is required for low-count interpretation [5]	[5]
Laboratory segregation and documentation for incompatible activities	Contamination prevention and monitoring principles are established in forensic QA [21,22]	[21,22]
Interpretive statement with limitations (fit-for-purpose)	Systematic review-level evidence warns against standalone conclusions and highlights comparability limits [3,4]	[3,4]

Minimum Contamination Controls (MCC)

A minimum contamination control set is justified because contamination sources have been empirically demonstrated at multiple workflow points, including consumables, and low-count findings are precisely where interpretive claims become highest-stakes [5,7,14]. While diatom testing is not DNA testing, established forensic contamination-control doctrine (e.g., dedicated areas, blanks across stages, monitoring, and documentation) provides transferable principles for structuring MCC [21,22].

Decision Algorithm: When to Order Diatom Testing and When It Adds Value

Step one emphasizes confirming the clinical-scene hypothesis space first, as diatom testing should be considered only after full scene information, circumstances, and autopsy findings are reviewed, given that drowning diagnosis remains multifactorial and no single test is definitive [1-3]. Step two requires explicitly defining the forensic proposition, ordering the test only if it addresses a specific question, such as antemortem aspiration versus postmortem immersion, or compatibility with site X versus site Y, rather than as a reflexive “drowning confirmation” [3,4]. Step three focuses on choosing samples based on the proposition and contamination risk, noting that if conclusions rely on closed organs, enhanced controls are necessary because low-count results are vulnerable to contamination and may be amplified by severe postmortem change [5,7,14]. Step four mandates requiring MCC and MRD before interpretation, with results reported as limited or uninterpretable for strong propositions if key blanks or controls, including consumable or slide blanks where relevant, are absent [5,7,21,22]. Step five concludes that interpretation should be supportive rather than standalone, integrating diatom findings with the totality of evidence and explicitly stating limitations related to comparability and contamination [3,4].

Future research agenda

Future work should prioritize multicenter inter-laboratory comparability studies, as systematic evaluation identifies insufficient comparability of quantitative and qualitative reporting as the strongest limitation, and multicenter designs offer the most direct path to actionable thresholds and error rates. In parallel, the development of shared reference materials and reporting standards, such as standard waters, seeded tissues, and harmonized reporting templates, would directly address the current fragmentation of protocols and outcome metrics. Validation must also extend across different water systems and stages of decomposition, since diatom communities vary by environment and postmortem change may alter detection in closed organs, requiring explicit stratification by water type and decomposition stage. Finally, critical reviews underscore taxonomy and identification variability as a major source of inconsistency, making closer collaboration with diatomologists a methodological necessity rather than an optional refinement (Table 6).

**Table 6:** Novelty map: how recent (2022–2025) evidence reshapes the diatom-test debate and enables a new comparative review structure

Key recent contribution	What it clarifies (and why it matters)	Remaining gap it exposes	How this review’s comparative frame leverages it (novel synthesis)
Medico-legal systematic review on diatom interpretation [3]	Summarizes why evidentiary value remains contested and interpretation is not standardized [3]	Lack of reproducible thresholds and contamination-aware interpretation frameworks [3]	Uses “mechanism plausibility ≠ evidentiary reliability” as the organizing principle for comparing methods and reporting standards
Narrative review of the long-standing diatom question [4]	Highlights persistent methodological heterogeneity and interpretive disagreements [4]	No consensus on tissue amounts, criteria, or standard protocols [4]	Positions microscopy and molecular approaches as different “targets” requiring different validity criteria
Newly identified disposable slide contamination [5]	Demonstrates that even single-use consumables can introduce diatoms [5]	Traditional QA checklists may miss hidden consumable contamination [5]	Expands contamination control discussion beyond classical labware/reagents to supply-chain QA and batch testing
Reused Kjeldahl flask contamination study [7]	Empirically links a specific labware practice to false positives [7]	Standard protocols can be undermined by underestimated, repeatable contamination sources [7]	Re-frames “false positives” as preventable system errors, not inevitable noise
Metagenomic diatom analysis for site inference [8]	Shows strengths/limits of broad-scale molecular inference (e.g., freshwater vs seawater distinctions) [8]	Precise geographic inference remains difficult even with extensive sampling [8]	Clarifies where DNA approaches add value versus where they risk overclaiming
DNA barcode-focused forensic paper [11]	Identifies barcode/library issues and mislabeling as central barriers [11]	Consensus barcode/reference standards are not yet mature [11]	Grounds the “cannot replace microscopy yet” argument in concrete library/ground-truth limitations
Practical microscopy simplification for drowning-site inference [13]	Proposes lower-magnification, trait-based classification with strong pilot performance claims [13]	Needs validation across diverse systems and jurisdictions [13]	Motivates a “tiered microscopy” discussion separating screening classification from high-rigor taxonomic ID

Limitations of this review

Several limitations should be acknowledged. First, this review adopts a narrative and critical approach rather than a fully systematic methodology; consequently, while the literature search was structured and targeted, it cannot be claimed that all potentially relevant publications were exhaustively identified. Second, the reviewed studies are highly heterogeneous with respect to study design, sample selection, analytical protocols, and reporting practices, which precludes quantitative synthesis and limits direct comparison of sensitivity, specificity, or diagnostic thresholds across studies. Third, the review relies on published data and reported methods, and therefore cannot independently verify primary laboratory procedures, contamination-control practices, or unpublished negative findings. Fourth, medico-legal interpretation of drowning and diatom evidence may vary across jurisdictions due to differences in legal standards, autopsy practices, and evidentiary thresholds, which may affect the generalizability of some conclusions. Finally, although this review emphasizes recent literature and methodological advances, ongoing developments in molecular techniques, reference databases, and contamination-control strategies may further modify the evidentiary landscape. The proposed interpretive framework should therefore be viewed as adaptive rather than definitive, intended to guide current forensic practice while remaining responsive to future validation and standardization efforts.

## Conclusions

Diatom evidence should be regarded neither as definitive proof nor as inherently unreliable in the diagnosis of drowning. Its medico-legal value depends fundamentally on whether the analytical method employed, the degree of contamination control, and the transparency of reporting are appropriate for the specific forensic proposition being addressed. Recent evidence demonstrating contamination from previously unrecognized sources, including disposable microscope slides, highlights that low-count diatom findings in closed organs are particularly vulnerable to false-positive interpretation unless supported by explicit control data.

A fit-for-purpose interpretive framework that explicitly links the forensic question, sample selection, analytical method, contamination safeguards, and reporting standards offers a pragmatic path forward. When applied selectively and interpreted within the context of full scene reconstruction and autopsy findings, diatom testing may contribute supportive information to drowning diagnosis and drowning-site inference. However, overstatement of its probative value in isolation risks misclassification and medico-legal error. Future progress in this field will depend on standardized reporting, validated contamination controls, and multicenter comparative studies capable of establishing reproducible interpretive thresholds.
